# Phenotypes Associated with NOTCH3 Cysteine-Sparing Mutations in Patients with Clinical Suspicion of CADASIL: A Systematic Review

**DOI:** 10.3390/ijms25168796

**Published:** 2024-08-13

**Authors:** Yuan Cao, Ding-Ding Zhang, Fei Han, Nan Jiang, Ming Yao, Yi-Cheng Zhu

**Affiliations:** 1 Department of Neurology, State Key Laboratory of Complex Severe and Rare Diseases, Peking Union Medical College Hospital, Chinese Academy of Medical Sciences and Peking Union Medical College, Beijing 100730, China; caoyuan0725@gmail.com (Y.C.); hanfei@pumch.cn (F.H.); drjnjiangnan@gmail.com (N.J.); 2 Central Research Laboratory, State Key Laboratory of Complex Severe and Rare Diseases, Peking Union Medical College Hospital, Chinese Academy of Medical Sciences and Peking Union Medical College, Beijing 100730, China; zhangdingding@pumch.cn

**Keywords:** CADASIL, NOTCH3, cysteine-sparing, clinical phenotype, radiological cerebral phenotype, systematic review

## Abstract

CADASIL (cerebral autosomal dominant arteriopathy with subcortical infarcts and leukoencephalopathy) is caused by *NOTCH3* mutations affecting the number of cysteines. The pathogenic role of cysteine-sparing *NOTCH3* mutations with typical clinical CADASIL syndrome is still debated. This review aimed to characterize *NOTCH3* cysteine-sparing mutations in patients with clinical suspicion of CADASIL. Articles on *NOTCH3* cysteine-sparing mutations with clinical suspicion of CADASIL were reviewed. Clinical and radiological cerebral phenotypes data were extracted and characterized across regions and compared with phenotypes of typical CADASIL patients. We screened 298 *NOTCH3* cysteine-sparing mutation individuals from 20 publications, and mutations in exon 3 were the most frequently reported (21.46%). Gait impairment (76.47%), cognitive impairment (67.47%), and stroke (62.37%) were the three most common clinical phenotypes; the most frequent radiological cerebral phenotypes were lacunes (74.29%) and cerebral microbleeds (72.73%). Compared with CADASIL patients, cognitive impairment and cerebral microbleed frequencies were significantly higher in patients with NOTCH3 cysteine-sparing mutations, while the white matter hyperintensities in anterior temporal polar and external capsule were rarely observed. Compared with Western patients, radiological phenotypes were more common than clinical phenotypes in cysteine-sparing Asian patients. More than half of cysteine-sparing patients had positive granular osmiophilic material deposits. *NOTCH3* cysteine-sparing mutations in patients with clinical suspicion of CADASIL mainly manifested with gait and cognitive impairment but rare white matter hyperintensities in anterior temporal pole and external capsule. Further studies are warranted to pay attention to atypical *NOTCH3* variants, which could guide specific diagnosis and help unravel underlying mechanisms.

## 1. Introduction

CADASIL, which stands for Cerebral Autosomal Dominant Arteriopathy with Subcortical Infarcts and Leukoencephalopathy, is the most frequently observed and widely recognized form of monogenetic hereditary cerebral small vessel disease [1,2]. It occurs due to over 220 mutations identified so far in the *NOTCH3* gene [2,3]. *NOTCH3* contains 33 exons and encodes a transmembrane receptor constituted by three domains. Of these, the extracellular domain (ECD) with 34 epidermal growth factor-like repeats (EGFrs) is the most critical contributor to maintaining protein tertiary structure [4]. A definitive diagnosis of CADASIL is established through genetic testing that identifies mutations in the *NOTCH3* gene, resulting in an abnormal number of cysteine residues (typically five or seven) in one of the EGFr domains of the NOTCH3 protein, which is particularly prominent in the vascular smooth muscle cells in the brain, confirming the consensus on CADASIL diagnosis [5]. NOTCH3 mediates the NOTCH signaling pathway, which is important in cell–cell communication and plays a major role in blood vessel development. Pathologically, the odd number of cysteines leads to NOTCH3 misfolding and ECD aggregation, which is considered as the primary pathogenic mechanism triggering cerebral vascular smooth muscle cytopathology [6]. Increasing studies have also identified cysteine-sparing mutations as mutations that do not affect the number of cysteines in patients with granular osmiophilic material (GOM) accumulation which consists of parts of NOTCH3 ECD by skin biopsy and is considered as the typical pathological finding of CADASIL [7,8]. Cysteine-sparing mutations could also trigger significant loss of structure in NOTCH3, which challenges the traditional standard of pathogenic mutations [9].

Clinical hallmarks for CADASIL suspicion are constantly facing challenges. Migraine with aura, acute encephalopathy, recurrent lacunar strokes, cognitive decline, gait, and mood disorders have long been recognized as typical clinical manifestations of CADASIL [1]. However, community-dwelling studies suggested that participants harboring *NOTCH3* cysteine-altering variants are associated with a comprehensive phenotypic spectrum, ranging from a severe CADASIL phenotype to non-penetrance [10]. Magnetic resonance imaging (MRI) features, including symmetrical subcortical white matter hyperintensities (WMHs) located in the anterior temporal lobe, and progressive lacunes, cerebral microbleeds, and enlarged perivascular spaces [11] can also be observed in other monogenic cerebral small-vessel diseases, increasing with age and disease severity [5]. Additionally, *NOTCH3* cysteine-sparing variants can also present with CADASIL-like clinical and radiological phenotypes, indicating their potential pathogenic role in CADASIL. These findings make it unclear whether *NOTCH3* cysteine-sparing mutations contribute to vasculopathy in patients with clinical suspicion of CADASIL.

Better characterization of *NOTCH3* cysteine-sparing mutations in patients with clinical suspicion of CADASIL can offer insights into the pathomechanisms underlying CADASIL and aid in managing affected individuals. Given the considerations mentioned above, in the present study, a systematic literature review was performed to summarize genetic mutations and clinical manifestations of reported individuals with *NOTCH3* cysteine-sparing nonsynonymous mutations, including missense, nonsense, and frameshift mutations, and compare both clinical and radiological phenotypes associated with those harboring typical mutations.

## 2. Results

### 2.1. Genetic Data and Patients

We included 20 publications from 298 identified for screening and extracted data on 268 *NOTCH3* cysteine-sparing mutations individuals with clinical suspicion of CADASIL (Supplementary Table S3) [4,12,13,14,15,16,17,18,19,20,21,22,23,24,25,26,27,28,29,30]. Genetic information was accessible for all the families involved in the study, with the exception of one family (consisting of five individuals), for which the precise mutation details were not disclosed, as the findings were only published in the form of a conference abstract.

**Figure 1:**
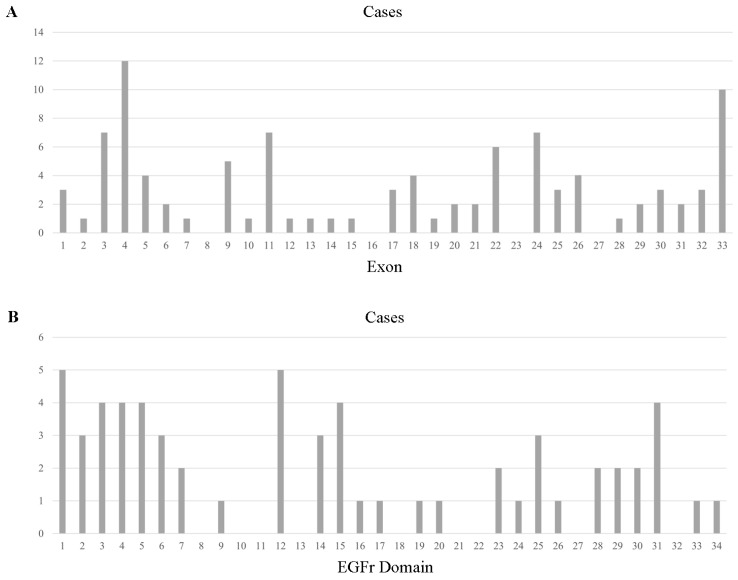
Cysteine-sparing case number across exons of *NOTCH3* (**A**) and EGFr domains of the NOTCH3 protein (**B**).

With respect to reported 100 different mutations in all the 263 individuals, a total of 95 mutations in 263 individuals were in the exon region, including 89 missense mutations, 5 frameshift mutations, and 1 nonsense mutation, distributed across the 28 exons (Figure 1). Exon 3 was the most frequently involved (57/254, 22.44%), with the p.R75P being the most common mutation (41/57, 71.93% of Exon 3 mutation). Mutations in Exon 4 type were the most abundant, with 12 different kinds of amino acid alteration and p.P167S being the most common one (15/31, 48.39% of Exon 4 mutation). Exon 24, 25, 5, 11, and 33 mutations were also notable, as shown in Supplementary Table S3. With regard to Exon 8, 16, 23, and 27, no mutations were reported from previous studies in the reviewed literature.

It is important to note that some cysteine-sparing mutations, including p.R75P, have been repeatedly confirmed as pathogenic in previous studies. However, it differs from the traditionally defined classic CADASIL pathogenic mutations as it does not alter the number of cysteines. Therefore, these mutations are still analyzed in this review, which can help us further explore the pathogenic mechanisms of *NOTCH3* cysteine-sparing mutations.

### 2.2. Frequency of Clinical Phenotypes

The average age at neurological deficits onset was 53.64 ± 2.32 (100/262). The clinical features of 248 identified patients are summarized, including stroke, cognitive impairment, psychiatric disturbance, gait impairment, headaches, and seizures (Table 1).

Clinical stroke attacks were reported in 62.37% (58/93) of patients with *NOTCH3* cysteine-sparing mutations. Ischemic events (including transient ischemic attack) were more common than hemorrhagic events, affecting 19 cases and 1 case, respectively. Cognitive impairment prevalence was reported in 13 published studies, ranging from 37.50% to 100.00%, without detailed information on severity, impaired domain, etc. Gait impairment was also commonly reported (13/17, from six studies). Headache, including migraine, was reported in 43.48% (40/92) of individuals. Most headache cases were not specified as migraine. The frequency of psychiatric disturbance, mainly specified as apathy or irritability, was reported as 38.96% (30/77). Thirty percent of individuals (9/30) were reported to have suffered a seizure or have epilepsy. Two studies reported that livedo reticularis was not noted in patients (0/5).

For cysteine-sparing mutations in Exon 1–6, 72.22% of individuals (78/108) had typical clinical CADASIL symptoms, while only 38.24% of individuals (26/68) had mutations in Exon 7–34 (Supplementary Table S3).

### 2.3. Frequency of Radiological Cerebral Phenotypes

Table 1 provides an overview of the primary neuroimaging features observed in *NOTCH3* cysteine-sparing mutations. This includes the involvement of white matter in the temporal pole and external capsule, the presence of lacunes, and cerebral microbleeds.

Ischemia was the most common radiological manifestation for individuals with *NOTCH3* cysteine-sparing mutations, and lacunes were noted in 74.29% (26/35). Cerebral microbleeds were also notable and present in 72.73% (40/55). Given that most publications were generally lacking in detail, location and multiple lesions were less clear. Of note, this sample with WMHs in the temporal pole and external capsule was relatively milder. Typical anterior temporal polar lesions were only present in 10.50% (23/219), and the external capsule was severely involved in 25.11% (58/231) of patients.

### 2.4. Comparisons of Phenotypes between Patients with NOTCH3 Cysteine-Sparing Mutations and Typical CADASIL

Typical manifestations of CADASIL often include recurrent lacunar strokes, cognitive deterioration, migraine with aura, mood disturbances, symmetrical periventricular WMHs without a clear cause, and a positive family history for similar symptoms. Based on previous studies and reviews, the prevalence of clinical manifestations was quite different between typical CADASIL with *NOTCH3* cysteine-altering mutations and suspected CADASIL with *NOTCH3* cysteine-sparing mutations (Table 2). Compared with 3252 CADASIL patients from 22 studies, the prevalence of stroke was almost the same as in patients with *NOTCH3* cysteine-sparing mutations (61.65% vs. 62.37%), and the prevalence of migraine (49.05% vs. 43.48%) as well. Interestingly, the proportion of cognitive impairment in patients with *NOTCH3* cysteine-sparing mutations was significantly more prevalent (67.47% vs. 35.54%), similar to psychiatric disturbance (38.96% vs. 32.95%).

WMHs in specific areas are highly suggestive and often considered a radiological hallmark for CADASIL, particularly in the anterior temporal lobe and external capsule. In nine studies (519 typical CADASIL patients) with specific mention of these two radiological features, the prevalence of WMHs in the anterior temporal lobe was 57.42% and that in the external capsule was 77.84%, which are significantly higher than those of patients with *NOTCH3* cysteine-sparing mutations (10.50% and 25.11%, respectively). In contrast, the lacunes and cerebral microbleeds in patients with *NOTCH3* cysteine-sparing mutations were more severe than those in typical CADASIL (74.29% vs. 62.00%, 72.73% vs. 35.80%, respectively), particularly for cerebral microbleeds with a significantly statistically higher proportion.

### 2.5. Differences between Asians and Patients in Western Countries

In comparison with patients with *NOTCH3* cysteine-sparing mutations from Western countries (Europe, North America, and Australia), several differences were observed in Asian patients. For mutations in different exons, *NOTCH3* mutations were more frequently detected in Exon 3 (23.98%), Exon 4 (10.05%), Exon 24 (9.59%), and Exon 25 (9.13%) in Asian populations; in contrast, for patients in Western countries, *NOTCH3* mutations were observed mostly in Exon 4 (21.43%), followed by Exon 3 (9.52%), Exon 9 (7.14%), and Exon 26 (7.14%) (Figure S1). Of note, p.R75P in Exon 3 was the leading mutation in Asians (40/53, 75.47%), particularly in South Korea and Japan.

The typical phenotypes of suspicious CADASIL with *NOTCH3* cysteine-sparing mutations varied significantly between ethnic groups (Table 3). A key factor contributing to these differences is the prevalence of Asian-specific CADASIL-causing mutations, such as p.R75P and p.R544C. For clinical phenotypes, Asians had a significantly lower proportion of headaches (29.58% vs. 66.67%), cognitive impairment (57.35% vs. 91.67%), and seizures (19.05% vs. 55.56%). Although proportions of stroke and psychiatric disturbance were not significantly different between the two groups, comparisons indicated that the stroke and psychiatric disturbance burden was lower in Asian patients (60.27% vs. 75.86%, 31.82% vs. 52.63%, respectively). In contrast, radiological cerebral phenotypes presented with more substantial burdens in Asians. In Asian patients, lacunae and cerebral microbleeds were twice as frequent as those in Western patients (88.00% vs. 40.00%, 80.44% vs. 33.33%, respectively). Similarly, Asian patients had a higher prevalence of anterior temporal pole and external capsule involvement with WMHs than those of European ancestry; however, no significant difference was found between these two groups (10.88% vs. 7.69%, and 26.39% vs. 6.67%, respectively). Notably, the presence of GOM deposits in skin biopsy was observed in 92.31% of Asian patients, significantly higher than in Western patients (42.86%).

## 3. Discussion

CADASIL diagnosis is definitively confirmed by *NOTCH3* cysteine-affecting mutations using genetic testing. However, patients with cysteine-sparing mutations who have typical clinical CADASIL features, extensive WMH, and even GOM deposits in skin biopsy are also highly suspected of CADASIL. In this study, we summarized the genetic spectrum of the *NOTCH3* cysteine-sparing mutations and found that mutations were more frequently detected in Exon 3, with p.R75P being the most common type. The overall burden of clinical phenotypes in patients with cysteine-sparing mutations is comparable with that in CADASIL subjects, except for a relatively milder involvement of WMHs in anterior temporal pole and external capsule. As a result, regional differences in genetic spectrums and clinical features are also observed, suggesting possible ethnicity-based mechanisms of *NOTCH3* cysteine-sparing mutations causing CADASIL.

### 3.1. Pathological Hallmarks and Molecular Insights

The accumulation and deposition of NOTCH3 ECD within the walls of small blood vessels are crucial pathological hallmarks of CADASIL. These phenomena are observed in patients and in a transgenic mouse model specifically designed to study this disease. For typical CADASIL patients, the odd number of cysteines may promote NOTCH3 ECD aggregation via their unpaired sulfhydryl groups [31]. The pathogenicity of cysteine-sparing mutations, including mutations outside EGFr, is still ambiguous. Some cysteine-sparing mutations, such as R75P, D80G, and A564T, showed significantly enhanced aggregation behavior in vitro similar to that of typical cysteine-affecting mutants associated with conformational changes in the protein [13,32,33]. Therefore, these mutations seem to cause a similar phenotype. For R61W and R213K, multimerization was absent and clinical symptoms were untypical [34,35]. According to these findings, additional mechanisms other than NOTCH3 ECD aggregation might be involved in *NOTCH3* cysteine-sparing mutations. Notch3 and other molecules/pathways have recently been supposed to underlie the pathogenesis of NOTCH-related diseases [36,37,38,39]. Although these mutations, such as R103X and L1515P, did not directly contribute to NOTCH3 protein accumulation, the canonical NOTCH3 signaling was dysregulated through destabilization of the heterodimer [36,39]. Additionally, an insertion mutation found within the intracellular domain of *NOTCH3* in a patient suspected of having CADASIL was anticipated to cause premature termination of the protein. This termination occurs without triggering the nonsense-mediated mRNA decay process, potentially impacting the downstream components of the Notch signaling pathway [40]. Notably, some other cysteine-sparing mutations without multimerization were still questionable about the possibility of polymorphism or other arteriopathy-causing mutations. Specifically, the mutations mentioned in the studies, p.Arg10Hisfs*16 [10], involving *NOTCH3* homozygous nonsense mutations are likely to lead to impaired NOTCH3 signaling. This could play a critical role in CADASIL progression. However, the contribution of loss-of-function effects of *NOTCH3* variants is still debated, and it remains unclear whether decreased NOTCH3 receptor activity also plays a central role. Delving into the functional characterization of these mutants could illuminate the pathogenetic mechanisms at the heart of CADASIL. This could provide deeper insights into how the disease develops and progresses.

### 3.2. Challenges in Confirming the Pathogenicity of Cysteine-Sparing NOTCH3 Mutations in CADASIL

Although cysteine-sparing NOTCH3 mutations have been studied more extensively, no consensus has been reached regarding their pathogenicity. Muiño reviewed 34 cases with a clinical suspicion of CADASIL and cysteine-sparing *NOTCH3* missense mutations and concluded the following criteria for pathogenic identification: (1) the patients had typical clinical CADASIL syndrome; (2) the patients had diffuse WMHs in MRI; (3) the study analyzed the 33 exons of the *NOTCH3* gene to rule out other pathogenic mutations; (4) the mutation had a MAF < 0.1%; and lastly (5) the patients had GOM deposits in the skin biopsy [4]. However, pathogenic criteria for clinical practice still face problems. Clinical and genetic diversity is a notable feature among CADASIL patients, with variations significantly influenced by the target population, study duration, and stage of the disease [41]. Conversely, the effectiveness of detecting GOM as a diagnostic marker for CADASIL is debatable. Recent findings have highlighted instances where GOM was absent in skin biopsies of CADASIL cohorts, particularly in patients carrying mutations in the EGFr 7–34 regions of the NOTCH3 gene. This inconsistency calls into question the reliability of GOM presence as a universal diagnostic criterion for CADASIL [42,43]. Therefore, the absence of GOM does not definitely exclude a CADASIL diagnosis, since genotype characteristics and different biopsy locations might greatly influence its positivity. In this review, participants with suspicious clinical CADASIL syndrome and a cysteine-sparing *NOTCH3* mutation were selected. According to MAF and database searching, mutations identified as polymorphisms were also excluded to collect data about potential pathogenic cysteine-sparing *NOTCH3* mutations associated with typical clinical CADASIL syndrome.

Muiño considered the cysteine-sparing *NOTCH3* missense mutations p.R61W, p.R75P, p.D80G, and p.R213K as potentially pathogenic. This indicates a belief that these specific genetic alterations could contribute to disease processes despite not affecting cysteine residues directly [4]. Consistently, these patients presented typical clinical CADASIL syndrome, MRI profiles, GOM deposits, and family history in our study. In addition, we also found that p.G73A, p.G73S, p.R107W, p.R75Q, p.A202V, p.G149V, p.H170R, p.D239N, p.V252M, p.E309K, p.P496L, p.A564T, p.V644D, p.D887A, p.T1098S, p.H1133Q, p.R1175W, p.G1347R, p.S1418L, p.R1761H, p.V1922L, and p.V1952M also presented typical CADASIL phenotypes, positive family history, or GOM deposits by skin biopsy except for some cases in which examination are not available (Supplementary Table S3). PolyPhen-2 predictions were conducted on the above variants, and all indicated probably damaging or possibly damaging mutations. Additionally, according to Muiño’s criteria, the newly identified mutations p.G73A, p.R75Q, p.S1418L, and p.R1761H in this study are potentially pathogenic. Based on the clinical data available in the literature, these mutations can be viewed as potential pathogenic mutations. This assessment suggests that they warrant additional investigation to provide mechanistic evidence of their role in disease processes.

### 3.3. Clinical Phenotypes of Cysteine-Sparing Mutations

The average age of presenting neurological symptoms or signs in CADASIL patients ranges from 45 to 51.3 years [30,44,45,46], specifically approximately 50 years for males and 53 for females [47], and that of *NOTCH3* cysteine-sparing carriers is 53.64 years in the current review. Given the fact that the a number of cases reported onset age, as well as the fact that limited information was provided on corresponding gender and onset symptoms, the actual age may be different.

Radiological findings in patients with *NOTCH3* cysteine-sparing mutations exhibit significant variability, reflecting a spectrum of clinical manifestations. WMHs in anterior temporal pole and external capsule are highly suggestive of CADASIL, observed in about 80% of all CADASIL patients [48,49]. Of patients with *NOTCH3* cysteine-sparing mutations, 91% did not have anterior temporal pole involvement [4]. Similarly, we found a lower proportion of anterior temporal pole WMHs (10.50%) and that of external capsule (25.11%). Altogether, these findings suggest that a relatively milder white matter involvement in these two specific locations might be a characteristic of *NOTCH3* cysteine-sparing mutations. The observed variation in temporal pole involvement between CADASIL cases with cysteine-sparing mutations and those with cysteine-affecting mutations could be attributed to the distinct convolutional structure and vascularization of the temporal pole. This area might be particularly susceptible to the effects of myelin depletion or edema, which can result from the aggregation of NOTCH3 ECD and changes in the microvasculature. These unique anatomical and physiological characteristics of the temporal pole could explain the differential impact observed in CADASIL phenotypes depending on the type of mutation [50]. In patients with cysteine-sparing mutations, these mutations are less involved in GOM deposits around vascular smooth muscle cells and may have less impact on the drainage of the interstitial fluid and white matter rarefaction. This hypothesis underscores the need for further research to validate the speculated effects and mechanisms. CMBs were prevalent in 72.73% of cases, with a marked increase in older patients, indicating a correlation between age and the burden of vascular lesions. The spatial distribution of CMBs, predominantly in the basal ganglia, thalamus, and brainstem, aligns with patterns seen in typical CADASIL but with potentially greater severity. Advanced MRI sequences and higher magnetic field strengths allowed for more sensitive detection of these lesions. However, variations in imaging protocols across studies may have contributed to inconsistencies in reporting, highlighting the need for standardized imaging criteria in future research.

The present result also suggests that compared with cysteine-sparing mutations in Exon 7–34, mutations in Exon 1–6 presented a high risk of typical CADASIL phenotype, somewhat in line with previous findings that the position of *NOTCH3* cysteine-affecting variants plays a crucial role in determining CADASIL clinical and radiological severity. Specifically, variants in EGFr 1–6 of *NOTCH3* are associated with an earlier onset of stroke and a more significant accumulation of WMHs, indicating a more severe disease progression [10,51,52,53]. Previous evidence speculated that *NOTCH3* EGFr 7–34 were likely associated with attenuated CADASIL or late-onset phenotype. Therefore, the manifestations were relatively mild at currently considered normal age for cerebrovascular disease [52]. Therefore, *NOTCH3* cysteine-sparing mutations located in the higher EGFr domains might represent an underrecognized risk factor for cerebral small vessel disease, suggesting the need for increased awareness and investigation into these variants’ impact on the disease’s pathology. Given the fact that the number of patients in different EGFr domains in the current sample was too low for further reliable analysis, we could not assess the associations with onset age, clinical phenotypes, and imaging features. Large-scale studies including more detailed phenotypic information are warranted.

Like typical CADASIL, the characteristics of clinical and radiological phenotypes vary across different regions in patients with *NOTCH3* cysteine-sparing mutations. The clinical manifestation burden of Asian patients was milder than Western patients; however, the radiological cerebral phenotypes of Asian patients were more notable, especially for lacunes and cerebral microbleeds. Previous research suggests that involvement of the white matter in the temporal pole was less common in Chinese and Korean populations, while TIA and stroke occurrences were more frequent in Chinese and Japanese populations. These groups are characterized by a high prevalence of the R544C mutation. This pattern hints at a potential mechanism influenced by varying genotype frequencies across different ethnicities, indicating that specific genetic variants like R544C might have distinct pathological impacts in diverse populations [54,55]. Additionally, vascular risk factors, including hypertension and diabetes mellitus, are more prevalent in Asians due to the notable lacune presence [56]. The radiological cerebral findings summarized here indicate that other genetic background effects should be considered as possible mechanisms.

Concerning limitations, the small sample size and a low number of *NOTCH3* cysteine-sparing mutants could skew the frequencies of different phenotypes. In addition, the number of studies in the Asian population is significantly higher than that in the Caucasian one. Moreover, due to lacking unified standards and definitions of various clinical phenotypes, the specific prevalence of sub-phenotypes, such as ischemic or hemorrhage stroke, migraine, and apathy, could not be evaluated further in our review. Finally, some population-based studies just analyzed the 33 exons of the *NOTCH3* gene, leaving the other atypical mutations such as small deletions or mutations involving the intron to be assessed.

### 3.4. Future Directions

Given the findings of this systematic review, several areas warrant further investigation. First, future studies should aim to standardize MRI protocols, including the use of specific sequences and magnetic field strengths, to ensure consistent detection and characterization of white matter lesions and vascular abnormalities across different patient populations. Advanced imaging techniques, such as high-resolution MRI and quantitative imaging biomarkers, could provide more detailed information about the extent and nature of vascular lesions in CADASIL. These techniques may help identify subtle changes not detectable with conventional imaging methods.

Longitudinal studies are needed to track radiological and clinical feature progression over time in patients with *NOTCH3* cysteine-sparing mutations. Such studies could provide insights into the natural history of the disease and identify early markers of disease progression.

Further research is needed to explore ethnic and geographic variability in the prevalence and phenotypic expression of *NOTCH3* cysteine-sparing mutations. Understanding these differences could inform region-specific diagnostic and management guidelines.

## 4. Materials and Methods

We registered a PREPARE (Practice guideline Registration for transPAREncy, No. IPGRP-2022CN186) at http://guidelines-registry.cn “http://guidelines-registry.cn/guid/1560?lang=en (accessed on 16 November 2023)” for the Chinese guideline for diagnosing and managing CADASIL. In addressing the challenges of diagnosing CADASIL, we adhered to the Preferred Reporting Items for Systematic Reviews and Meta-Analyses (PRISMA) guidelines to review literature on phenotypes linked to NOTCH3 cysteine-sparing mutations in individuals suspected of having CADASIL. Drawing on data from existing studies, this systematic review does not necessitate ethical standards committee approval. The authors confirm that all the supporting data can be found within the article and its Supplementary Materials.

### 4.1. Search Strategy

An extensive systematic literature search was conducted to identify all published CADASIL pedigrees, families, and individual patients up to September 2022 and was performed independently by three reviewers (C.Y., H.F., and J.N.). We searched the MEDLINE, EMBASE, China National Knowledge Internet, Wanfang Data, and the Cochrane Library using search strings that included the following terms: “CADASIL”, “Cerebral autosomal dominant arteriopathy with subcortical infarcts and leukoencephalopathy”, “cysteine”, “dementia”, “cognition”, “lacune”, “white matter”, “cerebral microbleeds”, “skin biopsy”, and “granular osmiophilic material”. The search string used for literature search is provided in Supplementary Table S1. The PICO (Population, Intervention, Comparison, Outcomes) framework was applied to structure our search strategy (Supplementary Table S2). We did not restrict the search by language; we limited it to human studies and included conference abstracts.

Two reviewers, C.Y. and H.F., independently evaluated the titles and abstracts of all identified publications in our search, without knowledge of each other’s decisions. Subsequently, full texts of studies selected at this initial phase were further examined for eligibility by these two reviewers, who also noted any reasons for exclusion. The inter-rater reliability was assessed using Cohen’s kappa coefficient to ensure consistency in study selection, and Cohen’s kappa was 0.92. Any discrepancies were addressed and resolved through discussion and agreement, involving a third reviewer (Y.M.) when necessary.

**Figure 2:**
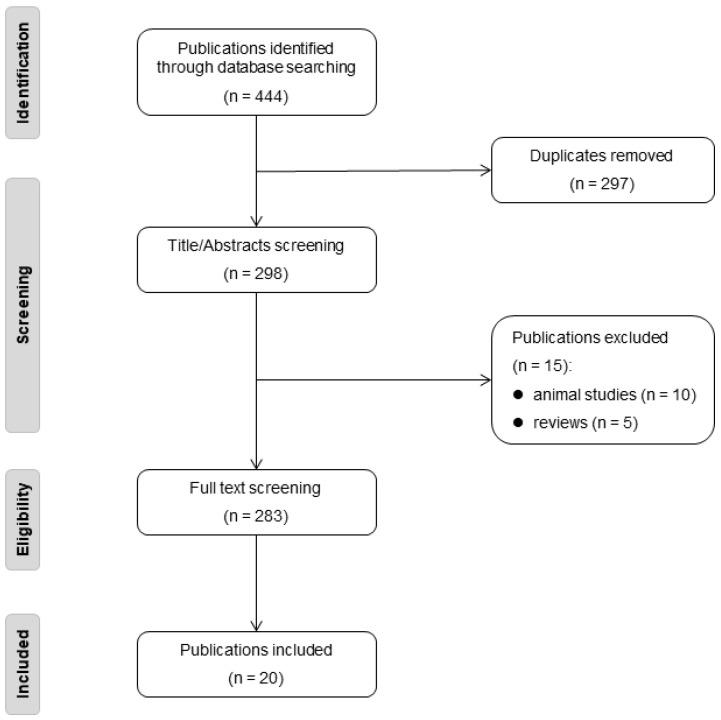
The flow diagram of literature review.

Our inclusion criteria encompassed studies presenting case reports, case series, or any other research design (excluding review articles) that described the clinical or cerebral radiological phenotype of one or more individuals. These descriptions ranged from simply noting an individual’s health status to providing detailed case reports. Furthermore, the study had to regard the rare variant as likely or definitively pathogenic. The process of our literature review is illustrated in the flow diagram provided in Figure 2.

### 4.2. Data Extraction

The reference lists of all included studies were screened to identify and assess other potentially relevant publications not captured in the initial search. The quality of included studies was assessed using the Newcastle–Ottawa Scale (NOS). Publication bias was evaluated through visual inspection of funnel plots and statistical tests where appropriate. We also assessed the quality of the conclusions drawn in the included studies to ensure the robustness of our findings.

We gathered information on the first author, year of publication, journal name, and the count of eligible individuals and pedigrees from each selected publication. For articles in languages other than English, we obtained a full translation whenever the English abstract failed to offer adequate details or was unavailable. We meticulously collected data for every individual deemed eligible using a pre-defined form. This included clinical cerebral phenotype aspects such as stroke, headache, cognitive symptoms, gait issues, seizures, and psychiatric conditions, as well as radiological cerebral phenotype elements like the occurrence of lacunes, white matter lesions, and cerebral microbleeds. These elements are recognized indicators of cerebral small vessel disease.

### 4.3. Data Synthesis

To evaluate the apparent frequency of each identified cerebral phenotype, both clinical and radiological, we compiled and summarized data concerning the presence or absence of these features. Additionally, we gathered cumulative evidence of any vascular radiological characteristics to provide a comprehensive overview of their occurrence. Specifically, descriptive statistics were used to summarize the clinical and demographic characteristics of the patients. For categorical variables, frequencies and percentages were calculated. Mean and standard errors (SEs) were reported for continuous variables. We analyzed the outcomes for patients with suspicious clinical CADASIL syndrome and a cysteine-sparing *NOTCH3* mutation, focusing on identifying common patterns and variations in the prevalence of associated phenotypes. This assessment aimed to elucidate the shared characteristics and distinctive features in the manifestation of these mutations. Specifically, the frequency of each clinical phenotype was calculated. Frequencies of phenotypes in *NOTCH3* cysteine-sparing mutation patients were compared with those of typical CADASIL patients using chi-square tests. Similarly, clinical and radiological phenotypes between Asian and Western patients were compared.

## 5. Conclusions

Patients with *NOTCH3* cysteine-sparing mutations manifested with typical clinical CADASIL syndrome and radiological profile, mostly with gait and cognitive impairment but rare WMHs in the anterior temporal pole and external capsule. The characterized phenotype summarized here could aid in further diagnosing and managing CADASIL-like patients with *NOTCH3* cysteine-sparing mutations. Evidence from clinical, radiological, and pathological GOM deposits indicated that most *NOTCH3* cysteine-sparing mutations have a phenotype similar to that of CADASIL. This implied the hidden pathogenesis of *NOTCH3* mutations, which needs further investigation.

## Figures and Tables

**Table 1:** Patient clinical and demographic information related to cysteine-sparing *NOTCH3* mutations.

Outcome	Study No.	Total Sample Size	Number of Outcomes	Percent of Outcomes	SE
Stroke	16	93	58	62.37%	0.0372
Cognitive impairment	13	83	56	67.47%	0.0272
Psychiatric disturbance	12	77	30	38.96%	0.0468
Gait impairment	6	17	13	76.47%	0.0619
Headaches	15	92	40	43.48%	0.0307
Seizures	6	30	9	30.00%	0.0652
WML (temporal pole)	16	219	23	66.67%	0.0210
WML (external capsule)	15	231	58	25.11%	0.0272
Lacunes	9	35	26	74.29%	0.0478
CMBs	10	55	40	72.73%	0.0456

WML, White matter lesions; CMBs, Cerebral microbleeds. SE, Standard error. Frequencies were compared using chi-square tests.

**Table 2:** Comparison of phenotypes between NOTCH3 cysteine-sparing mutations and typical mutations.

Phenotype	NOTCH3 Cysteine-Sparing Mutations	Typical CADASIL Mutations	*p* Value
Stroke	58/93 (62.37)	2005/3252 (61.65)	0.8894
Cognitive impairment	56/83 (67.47)	982/2763 (35.54)	<0.0001
Headaches	40/92 (43.48)	1389/2832 (49.05)	0.2930
Psychiatric disturbance	30/77 (38.96)	661/2006 (32.95)	0.2717
Anterior temporal pole WMHs	23/219 (10.50)	298/519 (57.42)	<0.0001
External capsule WMHs	58/231 (25.11)	404/519 (77.84)	<0.0001
Lacunes	26/35 (74.29)	47/76 (62.67)	0.1992
Cerebral microbleeds	40/55 (72.73)	213/595 (35.80)	<0.0001

CADASIL, cerebral autosomal dominant arteriopathy with subcortical infarcts and leukoencephalopathy; WMHs, white matter hyperintensities. Frequencies were compared using chi-square tests.

**Table 3:** Comparison of clinical and radiological cerebral phenotypes according to ancestry.

Phenotype	Asian Patients	Western Patients (Europe, North America, and Australia)	*p* Value
Stroke	44/73 (60.27)	22/29 (75.86)	0.1373
Headaches	21/71 (29.58)	20/30 (66.67)	0.0005
Cognitive impairment	39/68 (57.35)	22/24 (91.67)	0.0022
Psychiatric disturbance	21/66 (31.82)	10/19 (52.63)	0.0967
Seizures	4/21 (19.05)	5/9 (55.56)	0.0455
GOM deposits	12/13 (92.31)	9/21 (42.86)	0.0039
Anterior temporal pole WMHs	21/193 (10.88)	2/26 (7.69)	0.6186
External capsule WMHs	57/216 (26.39)	1/15 (6.67)	0.0885
Lacunes	22/25 (88.00)	4/10 (40.00)	0.0033
Cerebral microbleeds	37/46 (80.43)	3/9 (33.33)	0.0037

WMHs, white matter hyperintensities; GOM, granular osmiophilic material. Frequencies were compared using chi-square tests.

## Data Availability

The datasets used and/or analysed during the current study are available from the corresponding author on reasonable request.

## Supplementary Materials

The following supporting information can be downloaded at: https://www.mdpi.com/article/10.3390/ijms25168796/s1.

## Abbreviation List

**Abbreviation**	**Definition**
CADASIL	Cerebral Autosomal Dominant Arteriopathy with Subcortical Infarcts and Leukoencephalopathy
NOTCH3	Neurogenic Locus Notch Homolog Protein 3
ECD	Extracellular Domain
EGFr	Epidermal Growth Factor-like Repeats
GOM	Granular Osmiophilic Material
MRI	Magnetic Resonance Imaging
WMHs	White Matter Hyperintensities
CMBs	Cerebral Microbleeds
MAR	Missing at Random
MICE	Multiple Imputation by Chained Equations
SE	Standard Error
TIA	Transient Ischemic Attack
