# Supplementary material for: Phenotypes Associated with NOTCH3 Cysteine-Sparing Mutations in Patients with Clinical Suspicion of CADASIL: A Systematic Review
Source: Int J Mol Sci. 2024 Aug 13;25(16):8796. doi: 10.3390/ijms25168796 (PMC11354472; doi:10.3390/ijms25168796)
Supplement: Supplementary file 1 [file ijms-25-08796-s001.zip › ijms-3129956-supplementary.pdf]

Supplemental Materials

Table S1. Search Strategy with Boolean Operators.

Table S2. PICO Criteria.

Table S3. Summary of 267 *NOTCH3* cysteine-sparing mutations individuals.

Figure S1. Comparison of *NOTCH3* cysteine-sparing mutations in different exons between Asian and Western patients.

Table S1. Search Strategy with Boolean Operators.

1. ("CADASIL" OR "cerebral autosomal dominant arterio$ with subcortical infarct$ and leukoencephalopathy")
2. ("NOTCH3" OR "Notch?3" OR "Neurogenic locus notch homolog protein 3")
3. ("cysteine" OR "cysteine-sparing" OR "cysteine-involving")
4. 1 AND (2 OR 3)
5. ("stroke" OR "cerebrovascular disease" OR "brain ischemia" OR "intracranial arterial disease" OR "intracranial h?emorrhage")
6. ("dementia" OR "cognition" OR "cognitive")
7. ("gait" OR "movement")
8. ("headache" OR "migraine")
9. ("psychiatry" OR "mood" OR "apathy" OR "irritability")
10. 4 OR 5 OR 6 OR 7 OR 8 OR 9
11. ("white matter" OR "leukoaraiosis" OR "leukoencephalopathy$" OR "leukomalacia" OR "white matter hyperintensit$" OR "white matter hypodensit$" OR "WMH" OR "white matter magnetic resonance hyperintensit$")
12. ("lacune$" OR "lacunar" OR "infarction" OR "infarct$" OR "cerebral infarct$" OR "lacunar infarct$")
13. ("microbleed?" OR "cerebral microbleed?" OR "CMB" OR "hypointense lesion?" OR "hemosiderin" OR "cortical siderosis" OR "superficial siderosis")
14. 11 OR 12 OR 13
15. ("GOM" OR "granular osmiophilic material" OR "skin biopsy")
16. 4 AND 10
17. 4 AND 14
18. 4 AND 15
19. 16 OR 17 OR 18
20. REMOVE duplicates from 19
21. LIMIT 20 to humans

Table S2. PICO Criteria.

The PICO (Population, Intervention, Comparison, Outcomes) framework was applied to structure our search strategy:

- Population: Individuals with clinical suspicion of CADASIL.
- Intervention: Genetic testing identifying NOTCH3 cysteine-sparing mutations.
- Comparison: Clinical and radiological phenotypes compared with typical CADASIL mutations.
- Outcomes: Characterization of clinical and radiological phenotypes associated with NOTCH3 cysteine-sparing mutations.

Table S3. Summary of 267 *NOTCH3* cysteine-sparing mutations individuals.

Number	Mutation	Replaced	Substitution	Exon	Frequency	Typical Clinical CADASIL Syndrome	Family History	Whole Exon Analysis	GOM
**1**	p.L32fs	Leu		1	1	1	0	1	-*
**2**	p.M1L	Met	Leu	1	1	0	0	1	-
**3**	p.R10Hfs*16	Arg	His	1	2	2	2	0	0
**4**	p.R61W	Arg	Trp	2	2	2	2	1/1	1/2
**5**	p.D80G	Asp	Gly	3	2	2	2	2	1/2
**6**	p.G73A	Gly	Ala	3	2	2	1/1	1/2	1/1
**7**	p.G73S	Gly	Ser	3	1	1	-	0	-
**8**	p.R103X	Arg	*	3	1	1	-	-	0
**9**	p.R107W	Arg	Trp	3	1	1	1	0	-
**10**	p.R75P	Arg	Pro	3	41	41	20/23	2/40	3/3
**11**	p.R75Q	Arg	Gln	3	8	4/8	1/5	5/8	1/1
**12**	p.A198T	Ala	Thr	4	1	1	1	0	-
**13**	p.A202V	Ala	Val	4	1	1	-	0	-
**14**	p.D139N	Asp	Asn	4	1	0	1	1	-
**15**	p.D158N	Asp	Asn	4	2	0/2	0/2	2	-
**16**	p.G149V	Gly	Val	4	1	1	1	1	-
**17**	p.G172D	Gly	Asp	4	1	1	0	1	-
**18**	p.H170R	His	Arg	4	3	3	-	0/3	-
**19**	p.P167S	Pro	Ser	4	15	0	3/14	15	-
**20**	p.Q151E	Gln	Glu	4	2	2	1/1	0/2	-
**21**	p.R133S	Arg	Ser	4	1	1	1	0	1
**22**	p.R207H	Arg	His	4	1	1	1	0	-
**23**	p.R213K	Arg	Lys	4	2	2	2	0/1	-

**24**	c.680-8C>A	-	-	5	1	0	0	1	-
**25**	p.D239N	Asp	Asn	5	2	2	-	0/2	-
**26**	p.V237M	Val	Met	5	15	7/15	7/15	9/14	1/2
**27**	p.V252M	Val	Met	5	1	1	-	0	-
**28**	p.E309K	Glu	Lys	6	1	1	1	0	-
**29**	p.F292Y	Phe	Tyr	6	1	0	0	1	-
**30**	p.E362K	Glu	Lys	7	1	0	0	1	-
**31**	c.1379-5C>T	-	-	9	1	0	0	1	-
**32**	p.I470T	Ile	Thr	9	1	0	1	1	-
**33**	p.P477T	Pro	Thr	9	1	0	0	1	-
**34**	p.P496L	Pro	Leu	9	1	1	-	1	-
**35**	p.S497L	Ser	Leu	9	8	2/8	2/7	6/7	-
**36**	p.Q505H	Gln	His	10	2	0/2	1/2	2	-
**37**	p.A564T	Ala	Thr	11	2	2	2	0/2	2
**38**	p.H593R	His	Arg	11	5	0/5	2/5	5	-
**39**	p.L605V	Leu	Val	11	1	1	0	1	-
**40**	p.P572L	Pro	Leu	11	2	2	0/1	1/2	-
**41**	p.R592S	Arg	Ser	11	1	1	1	0	-
**42**	p.R607H	Arg	His	11	1	1	1	0	1
**43**	p.T577A	Thr	Ala	11	1	1	-	-	-
**44**	p.V644D	Val	Asp	12	1	1	1	0	-
**45**	p.G667S	Gly	Ser	13	1	0	0	1	-
**46**	p.G746S	Gly	Ser	14	1	0	0	1	-
**47**	p.P777A	Pro	Ala	15	1	0	0	1	-
**48**	c.2567-5C>T	-	-	17	2	0/2	0/2	2	-
**49**	p.D887A	Asp	Ala	17	1	1	1	1	-

**50**	p.G899D	Gly	Asp	17	1	1	0	1	-
**51**	p.D941E	Asp	Glu	18	3	0/3	0/3	3	-
**52**	p.G974E	Gly	Glu	18	1	0	0	1	-
**53**	p.H973Q	His	Gln	18	1	0	0	1	-
**54**	p.S978R	Ser	Arg	18	2	2	1/2	1/2	-
**55**	p.A1020P	Ala	Pro	19	2	2	2	-	1/2
**56**	p.R1100H	Arg	His	20	4	1/4	1/3	3/4	-
**57**	p.T1098S	Thr	Ser	20	1	1	1	0	1
**58**	p.H1133Q	His	Gln	21	1	1	-	0	-
**59**	p.R1175W	Arg	Trp	21	1	1	1	-	-
**60**	p.T1152M	Thr	Met	21	1	0	1	1	-
**61**	p.H1215L	His	Leu	22	1	1	0	-	0
**62**	p.H1235L	His	Leu	22	1	1	1	0	-
**63**	p.N1207S	Asn	Ser	22	1	0	1	1	-
**64**	p.P1166S	Pro	Ser	22	2	0/2	1/2	2	-
**65**	p.P1226S	Pro	Ser	22	2	0/2	0/2	2	-
**66**	c.3838-8A>T	-	-	24	1	0	1	1	-
**67**	p.A1314P	Ala	Pro	24	1	0	-	1	-
**68**	p.A1391P	Ala	Pro	24	1	0	-	1	-
**69**	p.A1450T	Ala	Thr	24	8	0/8	0/8	8	-
**70**	p.E1464fs	Glu		24	1	1	0	1	-
**71**	p.G1347R	Gly	Arg	24	8	1/8	3/8	7/8	-
**72**	p.S1418L	Ser	Leu	24	1	1	1	1	1
**73**	p.A1506T	Ala	Thr	25	2	0	0	1	-
**74**	p.L1515P	Leu	Pro	25	1	1	1	1	0
**75**	p.L1518M	Leu	Met	25	18	0/18	4/17	18	-

**76**	p.A1604T	Ala	Thr	26	3	3	3	3	-
**77**	p.A1850T	Ala	Thr	26	1	0	-	1	-
**78**	p.D1598V	Asp	Val	26	1	0	0	1	-
**79**	p.E1596D	Glu	Asp	26	1	0	0	1	-
**80**	p.N1588H	Asn	His	26	2	0/2	1/2	2	-
**81**	p.N1597K	Asn	Lys	26	1	0	0	1	-
**82**	p.A1729V	Ala	Val	28	1	0	0	1	-
**83**	p.R1761H	Arg	His	29	2	2	2	1/2	1/1
**84**	p.V1762M	Val	Met	29	1	1	1	1	0
**85**	p.A1850T	Ala	Thr	30	3	0/3	1/3	3	-
**86**	p.M1806fs	Met		30	1	1	0	1	-
**87**	p.A1927T	Ala	Thr	31	2	0/2	1/2	2	-
**88**	p.V1922L	Val	Leu	31	5	2/5	4/5	3/5	-
**89**	c.5816-9A>G	-	-	32	4	0	1/4	4	-
**90**	p.H1944Y	His	Tyr	32	1	1	0	1	-
**91**	p.V1952M	Val	Met	32	5	1/5	1/5	5	-
**92**	p.A2070V	Ala	Val	33	1	1	0	1	-
**93**	p.G2182D	Gly	Asp	33	1	0	0	1	-
**94**	p.L1976Pfs*11	Leu	Pro	33	1	1	0	1	-
**95**	p.L2294F	Leu	Phe	33	1	0	0	1	-
**96**	p.P2034A	Pro	Ala	33	1	1	0	1	-
**97**	p.P2209L	Pro	Leu	33	1	0	0	1	-
**98**	p.P2215A	Pro	Ala	33	2	0/2	0/2	2	-
**99**	p.R2022S	Arg	Ser	33	2	0/2	1/2	2	-
**100**	p.R2109W	Arg	Trp	33	1	0	0	1	-
**101**	p.T2270M	Thr	Met	33	1	0	0	1	-

*: - indicates not assessed.

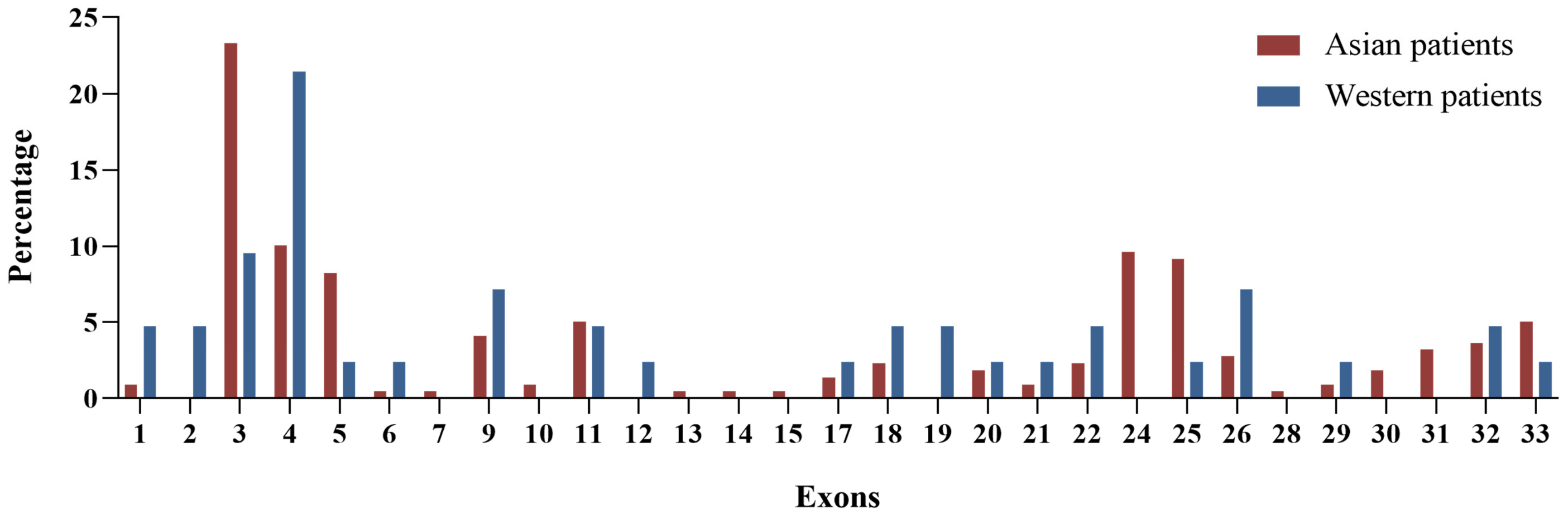

Figure S1. Comparison of *NOTCH3* cysteine-sparing mutations in different exons between Asian and Western patients.
